# Quality of Recovery after Laparoscopic Cholecystectomy Following Neuromuscular Blockade Reversal with Neostigmine or Sugammadex: A Prospective, Randomized, Controlled Trial

**DOI:** 10.3390/jcm10050938

**Published:** 2021-03-01

**Authors:** Jiwon Han, Ah-Young Oh, Yong-Tae Jeon, Bon-Wook Koo, Bo Young Kim, Donghyun Kim, Insung Hwang

**Affiliations:** 1 Department of Anesthesiology and Pain Medicine, Seoul National University Bundang Hospital, Gumi-ro, Bundang-gu, Seongnam-si 13620, Korea; hanjiwon@snubh.org (J.H.); ytjeon@snubh.org (Y.-T.J.); tendong2@gmail.com (B.-W.K.); to123215@gmail.com (B.Y.K.); flamethr@gmail.com (D.K.); Insung@snubh.org (I.H.); 2 Department of Anesthesiology and Pain Medicine, Seoul National University College of Medicine, Daehak-ro, Jongno-gu, Seoul 03080, Korea

**Keywords:** sugammadex, neostigmine, quality of recovery, laparoscopic cholecystectomy, patient-reported outcome

## Abstract

The risk of neuromuscular blockade is certainly minimized by sugammadex in combination with monitoring. However, the effect of sugammadex-aided recovery on patients’ satisfaction is unclear. This study compared the Quality of Recovery (QoR)-15 score, which is a patient-reported outcome, in patients undergoing laparoscopic cholecystectomy. Eighty patients were randomly assigned to the neostigmine or sugammadex groups. At the end of surgery, neostigmine or sugammadex was administered, and tracheal extubation was performed after confirmation of a train of four ratio ≥ 0.9. The QoR-15 questionnaire was administered at 1 day before surgery and on post-operative days (POD) 1 and 2. The primary outcome was the QoR-15 score on POD 1. The secondary outcomes were the QoR-15 score on POD 2, modified Aldrete score, length of post-anesthetic care unit stay, post-operative pain, administration of anti-emetics, urinary retention, and length of hospital stay. No significant differences were found in QoR-15 scores on POD 1 (94.4 vs. 95.5, *p* = 0.87) or 2 (116.3 vs. 122, *p* = 0.33). Secondary outcomes were also comparable, with the exception of urinary retention (15.8% neostigmine vs. 2.6% sugammadex, *p* = 0.04). This study demonstrated that the quality of recovery was comparable between the neostigmine and sugammadex groups when reversal and tracheal extubation were performed in accordance with the current guidelines.

## 1. Introduction

Recovery from surgery and anesthesia is a complex process that involves various physical and psychological changes. In addition to objective outcomes (e.g., laboratory values and rates of complications, morbidity, and mortality), each patient’s subjective experience and quality of life (e.g., pain, powerlessness, anxiety, and depression) have become important concerns. A validated, reliable tool for evaluation of patient-reported outcomes is the Quality of Recovery (QoR) questionnaire [1,2].

Laparoscopic cholecystectomy is the gold standard treatment for gallbladder disease and is one of the most commonly performed abdominal surgeries. Although it is a minimally invasive procedure, patients subsequently experience abdominal pain, shoulder tip pain derived from peritoneal stretching, and diaphragmatic irritation caused by the pneumoperitoneum [3,4]. In addition, the incidence of post-operative nausea and vomiting is higher than the incidence encountered in other surgeries [5].

A Cochrane systematic review revealed significantly fewer composite adverse events (e.g., lower risk of bradycardia, post-operative nausea and vomiting, and overall signs of post-operative residual paralysis) in patients who received sugammadex, compared with patients who received neostigmine [6]. However, findings regarding long-term patient outcomes have been controversial. The POPULAR (Post-anaesthesia pulmonary complications after use of muscle relaxants) study reported that the use of neuromuscular blocking agents was associated with an increased risk of post-operative pulmonary complications; the use of reversal agents did not reduce this risk [7]. In contrast, the recent STRONGER (Sugammadex versus neostigmine for reversal of neuromuscular blockade and postoperative pulmonary complications) study demonstrated a significantly lower incidence of major pulmonary complications after the use of sugammadex [8]. Another study showed that the use of sugammadex reduced 30-day unplanned readmission, length of hospital stay, and related hospital charges, compared with neostigmine [9]. Despite these studies of sugammadex usage, only a few studies have focused on patient-reported outcomes.

In this prospective, randomized, controlled study, we hypothesized that sugammadex would be superior to neostigmine in patient’s recovery, so we evaluated post-operative patient satisfaction following the reversal of neuromuscular blockade (NMB) with neostigmine or sugammadex using the QoR-15 score among patients undergoing laparoscopic cholecystectomy.

## 2. Materials and Methods

This prospective randomized controlled trial was approved by the Institutional Review Board at Seoul National University Bundang Hospital, Seongnam, South Korea (IRB approval number: B-1910/571-004; chairperson: Prof Hak Chul Jang, approval date: 8 January 2020) and registered in ClinicalTrials.gov (NCT 04332627). This manuscript adheres to the applicable CONSORT guidelines. Written informed consent was obtained from all participants.

Eighty patients (aged 20–70 years) scheduled to undergo laparoscopic cholecystectomy were enrolled in this study. Patients who had renal dysfunction, an allergy to a study drug, neuromuscular disease, cognitive dysfunction, or inability to respond to the questionnaire, as well as those who refused to participate, were excluded from the study.

Enrolled participants were randomly assigned to the neostigmine or sugammadex group at 1:1 ratio using a web-based randomization code. The assignments were placed in a concealed envelope and opened by an anesthesiologist who performed anesthesia management, prepared and administered study drugs; that anesthesiologist was not involved in the questionnaire survey.

Routine monitoring (e.g., pulse oximetry, non-invasive blood pressure, electrocardiography, capnography, and esophageal temperature assessment) was performed in the operating room. The patient’s right arm was fixed to the arm board at the right angle, and the remaining four fingers were fixed at the arm board for free movement of thumb. Neuromuscular monitoring was performed by stimulation of the ulnar nerve and measurement of adductor pollicis muscle movement by acceleromyography (Phillips NMT modular unit, Phillips Healthcare, Amsterdam, The Netherlands). Anesthesia was induced with 1.5 mg kg^-1^ propofol and remifentanil; it was maintained with desflurane and target-controlled infusion of remifentanil using the Orchestra infusion pump system (Fresenius vial, Brezins, France). After loss of consciousness had been confirmed, the NMT module was calibrated, and 0.6 mg kg^−1^ rocuronium was administered. The endotracheal tube was inserted after relaxation had been confirmed by train-of-four (TOF) count to be 0. If necessary, an additional 5 or 10 mg rocuronium was administered during surgery.

At the end of surgery, neostigmine or sugammadex was administered in accordance with guidelines [10]. In the neostigmine group, when the TOF count was < 2, administration was postponed until a TOF count of 2 was detected. When the TOF count was ≥ 2 and the TOF ratio was < 0.4, 50 µg kg^−1^, neostigmine was administered; when the TOF ratio was 0.4–0.9, 20 µg kg^−1^ neostigmine was administered. For all patients, 0.4 mg glycopyrrolate was co-administered with neostigmine. In the sugammadex group, when the post-tetanic count was ≥ 1 and no TOF response was detected, 4 mg kg^−1^ sugammadex was administered; when the TOF count was ≥ 1, 2 mg kg^−1^ sugammadex was administered. The endotracheal tube was removed after confirmation of a TOF ratio ≥ 0.9. Following confirmation of adequate respiration and vital signs, all participants were transferred to the post-anesthesia care unit (PACU).

For the pain management, i.v. 50 µcg of fentanyl was administered as a primary rescue analgesic, followed by an additional 50 µcg of fentanyl and 30 mg of ketorolac at PACU. In the general ward, patients were treated with p.o. tramadol 37.5 mg/acetaminophen 325 mg tid from the time of sips of water. If bowel function was not yet recovered, or pain could not be controlled by oral medication, nalbuphine 10 mg, pethidine 25 mg, tramadol 100 mg, and morphine 5 mg were administered in sequence. If patients had side effects of opioid, ketoprofen 100 mg or propacetamol 1 g were administered. For the management of postoperative nausea and vomiting, ramosetron 0.3 mg was administered as the primary rescue anti-emetics, followed by metoclopramide 10 mg and palonosetron 0.075 mg.

The quality of recovery was assessed using the QoR-15 questionnaire, which was validated and reliably translated into a Korean version [11,12]. Each item was rated on an 11-point scale (0–10), with responses that ranged from ‘none of the time’ to ‘all of the time’. The total score on the QoR-15 ranged from 0 (poorest quality of recovery) to 150 (best quality of recovery) (Appendix A, Appendix B). The questionnaire was administered three times (1 day before surgery and on post-operative days 1 and 2 [POD1 and POD2]) by researchers who were unaware of the group assignments. If the participant was discharged or recovered on a weekend, a mobile questionnaire with the same content was used, rather than direct patient visits.

The primary endpoint of the study was the global QoR-15 score on POD1. The scores on POD2 and mean changes in global scores between assessments performed pre-operatively and on POD1 and POD2 were also analyzed. The following additional data were collected: the modified Aldrete score (i.e., five items from 0 to 2 points: consciousness, mobility, breathing, circulation, and color; global scores of 0–10, where higher score indicates greater likelihood of recovery) at PACU arrival; length of stay in the PACU (minutes); post-operative pain measured by an 11-point numerical rating scale at 30 min, 6 h, and 24 h after surgery; number of doses of rescue analgesics and anti-emetics; morphine equivalent dose of opioid rescue analgesics; post-operative urinary retention diagnosed by bladder ultrasonography in the general ward; and length of hospital stay. From the time of administration of the study drugs, side effects including allergic reaction and hemodynamic change were monitored. We evaluated for respiratory depression and muscle weakness during PACU stay using modified Aldrete score. Any complications were evaluated up to discharge.

### Statistical Analyses

Sample size was calculated based on previously reported values of the mean and standard deviation QoR-15 score in patients after general anesthesia and intermediate surgery (114 ± 18); an average difference of ≥ 12 in the global QoR-15 score was considered a clinically significant improvement [13] using web based sample size calculator. The estimated sample size was 40 participants per group with a power of 0.8, type 1 error of 0.05, and dropout rate of 10%. Continuous variables were compared using Student’s *t*-test or the Mann–Whitney U test and are presented as mean ± standard deviation. Categorical variables were compared using the x^2^ test and are presented as number (proportion, %). Statistical analyses were performed using SPSS software (SPSS Inc., Chicago, IL, USA) and *p*-values < 0.05 were considered statistically significant.

## 3. Results

**Figure 1:**
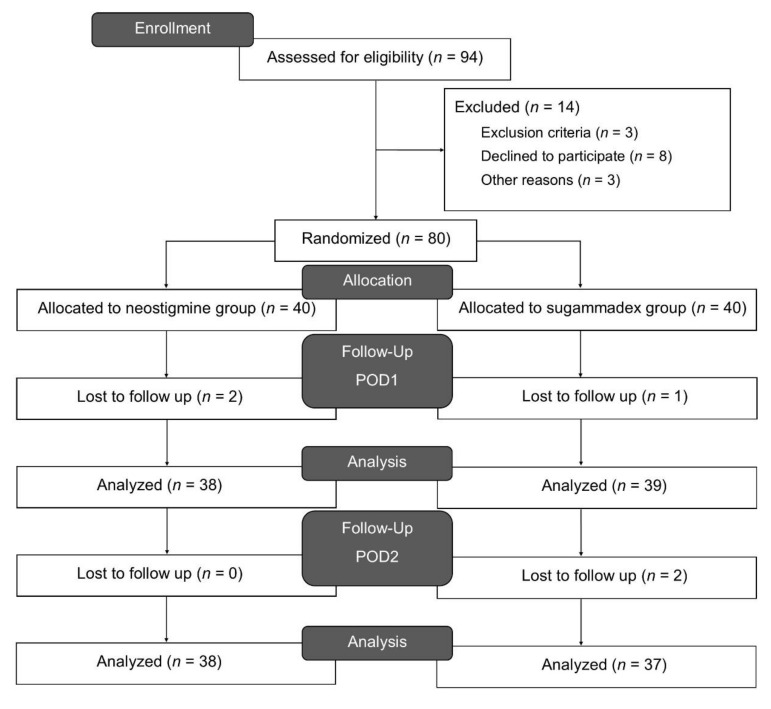
Consort flow diagram.

This study was conducted between February 2020 and May 2020. Ninety-four patients were assessed for eligibility and 14 patients were excluded for the following reasons: one had end-stage kidney disease, one had amyotrophic lateral sclerosis, one had cognitive dysfunction due to intracranial hemorrhage history, eight refused to participate, and three were excluded for other reasons. Forty participants were enrolled in each group. Among the enrolled patients, two in the neostigmine group and one in the sugammadex group did not respond on POD1, while two in the sugammadex group did not respond on POD2 (Figure 1). All of the non-response cases were patients who received a mobile questionnaire. The baseline demographic and clinical characteristics of the groups were comparable (Table 1). The average dose of rocuronium administered was 41.1 ± 8.2 mg, neostigmine was 3 ± 1.1 mg, and sugammadex was 161.1 ± 56.2 mg.

**Figure 2:**
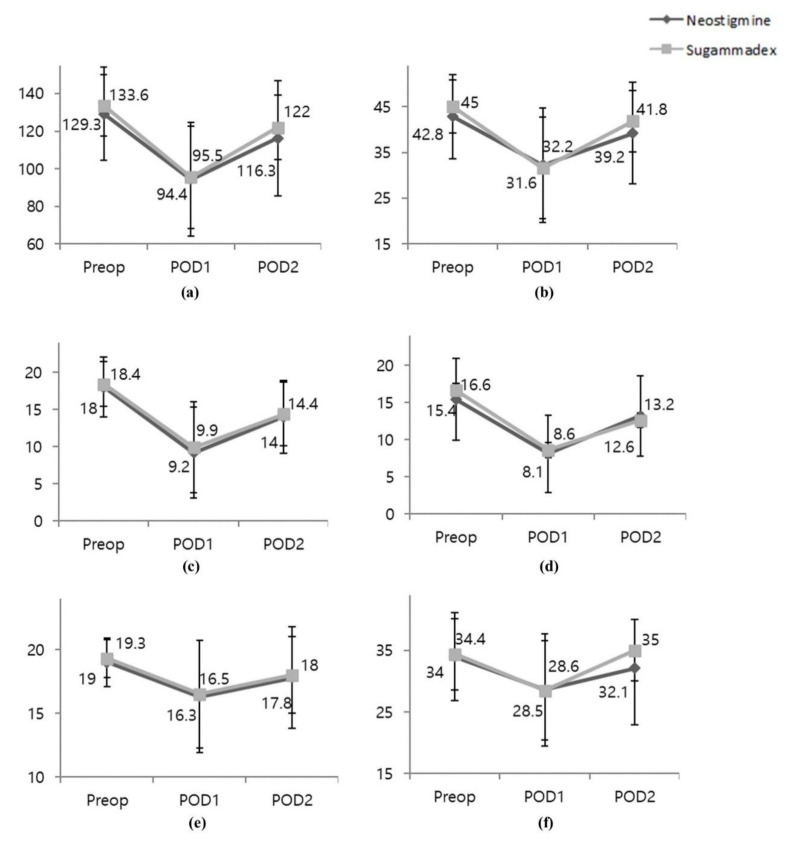
Quality of Recovery-15 at pre-operative and post-operative 1 and 2 days. (**a**) Global scores; (**b**) Physical comfort; (**c**) Physical independence; (**d**) Pain; (**e**) Physical support; (**f**) Emotional state. Data are mean with error bars showing SD. Neostigmine (dark gray rombus); Sugammadex (light gray square). There were no significant differences in QoR-15 global or sub-scores between neostigmine and sugammadex groups.

The overall baseline pre-operative global QoR-15 score was 131.5 (21.2) and decreased 27.6% to 95.0 (29.0) on POD1 and 9.4% to 119.1 (25.3) on POD2. The overall and subgroup scores and individual scores of physical comfort, physical independence, pain, psychological support, and emotional state did not differ between groups (Figure 2, Appendix C). Post-operative recovery profiles (e.g., modified Aldrete score, length of PACU stay, post-operative pain scores, administration of rescue analgesics, rescue anti-emetics and length of hospital stay) were comparable between the groups; however, the incidence of post-operative urinary retention was significantly lower in the sugammadex group (Table 2).

No symptoms or signs of adverse effects of the study drugs (e.g., allergic reaction) were observed in either group during the study period. However, one patient underwent ERCP (Endoscopic retrograde choangiopancreatography) due to persistent severe pain and fever after surgery. Another patient suffered pleural effusion and atelectasis after surgery. Both two patients were in the neostigmine group. There was no case of mortality or catastrophic complications.

## 4. Discussion

This study showed that patient-reported QoR-15 scores were comparable between the sugammadex and neostigmine groups when the entire process was performed in accordance with current guidelines (i.e., proper dose of reversal agent under the qualitative neuromuscular monitoring and tracheal extubation after confirmation of a TOF ratio ≥ 0.9).

A patient’s perception of their health status and quality of life is one of the most important outcome measures. The QoR-40, which was first developed by Myles in 2000, consisted of 40 questions in five dimensions: physical comfort, physical independence, pain, psychological support, and emotional status [1]. The QoR-40 is a validated, reliable tool to measure the quality of recovery after anesthesia and surgery [2,14]. In 2013, a shortened version of the QoR-40 (i.e., the QoR-15) was developed, which consists of 15 questions; the average time to complete the questionnaire was reduced from 6.3 min to 2.5 min, while validity and reliability were maintained [15,16]. In this study, the QoR-15 was used instead of the QoR-40 to increase the response rate by reducing the burden on patients; it also was expected to reduce the bias related to non-responding patients.

Previous investigation regarding the effects of reversal agents on post-operative recovery using a post-operative quality of recovery scale reported that sugammadex was superior only in the immediate post-operative physiological or nociceptive domains, whereas there were no differences in overall recovery and POD1 scores [17,18]. We did not perform an assessment of the immediate post-operative period because the superiority of sugammadex during that period has been established in prior studies. Additionally, the QoR scale is unique in that its results are entirely determined by patient responses, while the post-operative quality of recovery scale includes physician-assessed factors.

Sugammadex reduces the incidence of residual NMB, which is associated with the occurrence of critical respiratory events during the early post-operative recovery period that may affect long-term outcomes [6,19,20]. However, we administered appropriate doses of both reversal agents according to the NMB state, as confirmed by a qualitative nerve stimulator response and TOF ratio ≥ 0.9 before tracheal extubation. Hence, our results might have been more strongly affected by inherent characteristics of neostigmine and sugammadex, rather than the incidence of residual NMB.

However, based on the findings in previous reports, sugammadex may have exhibited a beneficial effect on the respiratory system, compared with neostigmine. We previously showed that, when reversal agents were administered in accordance with NMB status confirmed by a qualitative nerve stimulator response, but without confirming TOF ratio ≥ 0.9 before tracheal extubation, the incidence of residual NMB upon arrival in the PACU was 44% after neostigmine, whereas it was 0% after sugammadex. However, all patients who showed residual NMB recovered to TOF ratio ≥ 0.9 within 15 min [21]. Additionally, electromyographic activity of the diaphragm and intercostal muscles, PaO_2_, and tidal volume increased after sugammadex, compared with neostigmine, even though the TOF ratios were comparable between groups [22,23]. However, in order to obtain these advantages of sugammadex, it must be based on proper monitoring and adequate dosages.

Other factors may have had greater impacts on the QoR score, compared with the type of reversal agent. Score differences have been reported according to cholecystectomy indication; patients who were diagnosed with acute cholecystitis or pancreatitis scored lower than those with chronic cholecystitis, gallbladder polyps, or stones. Additionally, the QoR score has a sex-specific difference; women had lower mean QoR scores than men [24].

It was somewhat interesting that the incidence of post-operative urinary retention was lower in sugammadex group. This finding is presumably related to the co-administration of anti-cholinergics (e.g., atropine or glycopyrrolate) to prevent the cholinergic side effects of neostigmine. Anti-cholinergic drugs antagonize post-junctional muscarinic receptor, which results in inhibition of bladder destrusor muscle contraction and obstruction of the urinary outlet [25,26]. In this study, neostigmine was always administered with 0.4 mg glycopyrrolate, whereas the sugammadex group did not receive glycopyrrolate. Our results are consistent with the findings of a previous retrospective study, which revealed that the incidence of urinary retention was lower in a group of patients who received sugammadex following total knee replacement surgery [27]. Additionally, the mean age of patients with and without urinary retention was comparable (53.7 ± 8.2 vs. 52.5 ± 11.4, *p*-value = 0.807). It could be interpreted that the use of anticholinergics had a greater effect on the occurrence of postoperative urinary retention rather than age. Future investigations are needed to clarify the relationship between urinary retention and sugammadex or neostigmine-glycopyrrolate administration in various populations and during different surgeries.

There are limitations in this study. Neuromuscular management under research conditions may differ from real-word clinical practice. Indeed, proper neuromuscular monitoring is rarely implemented in many clinical practice situations. In two recently published large scale multi-center cohort analyses (i.e., the POPULAR and STRONGER studies), the rates of neuromuscular monitoring were 40% and 64%; moreover, tracheal extubation after confirmation of a TOF ratio ≥ 0.9 was performed in only 16.5% of patients [7,8]. Further investigations in real-world clinical practice are needed. Second, our study’s time frame was too late to detect residual neuromuscular blockade and too early to detect other surgical or medical complications. Third, the mobile QoR questionnaire was replaced instead of visiting the patients in case of patients’ discharge or recovery at weekend, the difference between the face-to-face and remote questionnaires can be a confounding factor. Lastly, it would have been better if we had investigated the state of neuromuscular blockade at emergence and recovery time.

## 5. Conclusions

In conclusion, there was no significant difference in the quality of recovery scores on POD1 or POD2, as measured by the QoR-15, when sugammadex or neostigmine was used for reversal of NMB in patients undergoing laparoscopic cholecystectomy with reversal and tracheal extubation performed in accordance with the current guidelines.

## Figures and Tables

**Table 1:** Characteristics of patients receiving neostigmine or sugammadex for laparoscopic cholecystectomy.

	Neostigmine, *n* = 38	Sugammadex, *n* = 39
Age (years)	51.3 ± 11.5	54.1 ± 10.7
Weight (kg)	69.1 ± 12.5	68.1 ± 15.5
Height (cm)	164.5 ± 9.1	166.6 ± 8.6
Sex; male	18 (47.4%)	22 ± 56.4%
Operation time (min)	41.2 ± 19.2	43.0 ± 21.2
Anesthesia time (min)	58.3 ± 22.2	63.6 ± 22.6
Diagnosis		
Chronic cholecystitis	16 (42.1%)	23 (59%)
Acute cholecystitis	12 (31.6%)	10 (25.6%)
Acute pancreatitis	2 (5.3%)	0 (0%)
GB Polyp	6 (15.8%)	5 (12.8%)
GB Adenomatosis	1 (2.6%)	1 (2.6%)
GB stone	1 (2.6%)	0 (0%)
ASA physical status		
1	18 (47.4%)	15 (38.5%)
2	18 (47.4%)	19 (48.7%)
3	2 (5.3%)	5 (12.8%)
Hypertension	12 (31.6%)	9 (23.1%)
Diabetes mellitus	4 (10.5%)	6 (15.4%)
Coronary artery disease	3 (7.9%)	8 (20.5%)
Cerebral vascular disease	1 (2.6%)	2 (5.1%)
Cancer	6 (15.8%)	10 (25.6%)

Values are presented as mean ± SD or number (proportion). Abbreviation: GB, gall bladder; ASA, American Society of Anesthesiologists.

**Table 2:** Post-operative recovery profiles.

	Neostigmine, *n* = 38	Sugammadex, *n* = 39	*p*-Value
Modified Aldrete score	7.5 ± 0.6	7.4 ± 0.5	0.48
PACU stay (min)	27.7 ± 8.7	29.8 ± 9.4	0.32
Pain score (NRS)			
30 min	6.5 ± 1.4	6.6 ± 1.2	0.76
6 h	3.6 ± 1.1	3.4 ± 1.0	0.41
24 h	3.0 ± 0.7	2.9 ± 0.6	0.22
Rescue analgesics *	3.2 ± 1.9	3.3 ± 1.2	0.79
Morphine equivalent dose (mg)	14.5 ± 7.3	15.6 ± 6.2	0.46
Rescue anti-emetics *	0.4 ± 0.7	0.4 ± 0.7	0.92
Urinary retention	6 (15.8%)	1 (2.6%)	0.04 ^†^
Length of hospital stay (days)	3.5 ± 0.95	3.7 ± 1.0	0.29

Values are presented as mean ± SD or number (proportion). Abbreviation: PACU, post-anesthetic care unit; NRS, numerical rating scale * Average number of administration per person. ^†^ *p* < 0.05.

**Figure A1:**
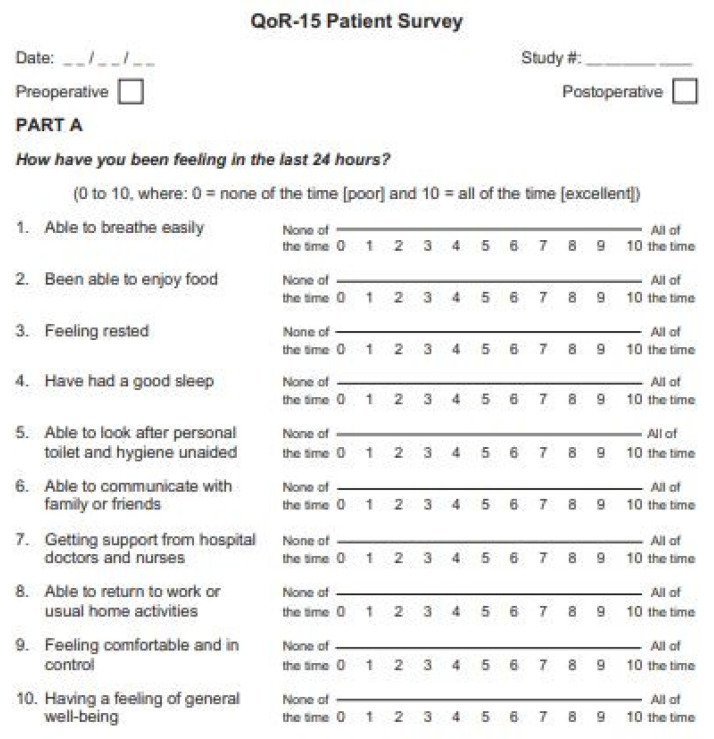

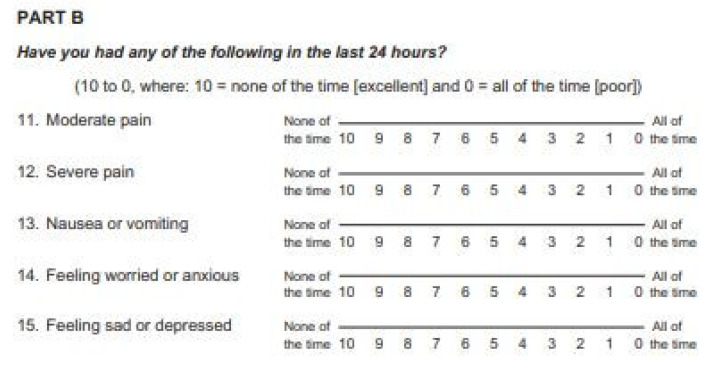
The Korean version of Quality of Recovery-15 questionnaire. The shortened version of quality of recovery-15 questionnaire, date, operation date, number of patient, Read the following questions and mark the applicable scores. I. How have you been feeling in the last 24 hours? 0 = none of the time, 10 = all of the time. II. Have you had any of the following symptoms in the last 24 hours? 10 = none of the time, 0 = all of the time.

## Data Availability

The raw data of this research will be available by the first author J.H., to any qualified researcher upon reasonable request.

## Appendix A

**Table A1:** Quality of Recovery (QoR)-15 questionnaire item.

1.	Able to breathe easy
2.	Been able to enjoy food
3.	Feeling rested
4.	Have had a good sleep
5.	Able to look after personal toilet and hygiene unaided
6.	Able to communicate with family or friends
7.	Getting support from hospital doctors and nurse
8.	Able to return to work or usual home activities
9.	Feeling comfortable and in control
10.	Having a feeling of general well-being
11.	Moderate pain
12.	Severe pain
13.	Nausea or vomiting
14.	Feeling worried or anxious
15.	Feeling sad or depressed

## Appendix C

**Table A2:** The global and sub-scores of QoR-15 questionnaires.

	Neostigmine		Sugammadex				*p*-Value		
	Preop *n* = 38	POD1 *n* = 38	POD2 *n* = 38	Preop *n* = 39	POD1 *n* = 39	POD2 *n* = 37	Preop	POD1	POD2
Physical comfort									
Breath	9.7 ± 0.7	8.2 ± 2.7	8.7 ± 2.4	9.6 ± 1.0	7.5 ± 2.6	9.4 ± 1.0	0.73	0.21	0.11
Eat	8.0 ± 3.3	5.2 ± 4.0	7.0 ± 3.0	8.9 ± 2.1	5.1 ± 3.9	7.7 ± 2.5	0.19	0.84	0.31
Rest	8.8 ± 2.0	5.7 ± 3.3	7.9 ± 2.6	9.3 ± 1.4	6.4 ± 3.0	8.5 ± 1.5	0.20	0.33	0.21
Sleep	8.3 ± 2.3	5.9 ± 3.2	7.7 ± 2.6	8.3 ± 2.1	6.1 ± 3.0	8.5 ± 1.4	0.97	0.86	0.13
Nausea	8.1 ± 2.9	7.1 ± 3.5	7.8 ± 3.2	8.9 ± 2.5	6.7 ± 3.4	7.7 ± 3.0	0.21	0.56	0.93
Physical independence									
Wash	9.3 ± 1.5	6.2 ± 3.6	8.2 ± 2.5	9.6 ± 1.0	6.1 ± 3.6	8.3 ± 1.9	0.35	0.82	0.79
Work	8.7 ± 2.7	3.0 ± 3.5	5.9 ± 3.5	8.8 ± 2.4	3.8 ± 3.5	6.1 ± 3.0	0.12	0.30	0.72
Pain									
Moderate pain	7.1 ± 3.1	3.2 ± 3.0	6.1 ± 2.6	8.0 ± 2.3	3.6 ± 3.0	5.5 ± 2.3	0.16	0.51	0.34
Severe pain	8.3 ± 2.8	4.9 ± 2.9	7.1 ± 3.2	8.6 ± 2.3	5.0 ± 3.1	7.1 ± 2.2	0.67	0.94	0.96
Psychological support									
Talk	9.5 ± 1.1	7.3 ± 3.0	8.7 ± 2.2	9.6 ± 1.1	7.4 ± 2.9	9.0 ± 1.6	0.58	0.92	0.45
Help	9.6 ± 1.0	9.0 ± 2.0	9.1 ± 2.0	9.7 ± 0.6	9.1 ± 1.8	9.0 ± 2.0	0.39	0.77	0.78
Emotional state									
Emotion	9.2 ± 1.4	7.7 ± 2.8	8.5 ± 2.4	9.3 ± 1.3	8.4 ± 1.9	9.0 ± 1.3	0.62	0.17	0.22
Well-being	8.7 ± 2.0	5.8 ± 3.0	7.6 ± 2.7	8.5 ± 1.6	5.9 ± 2.7	8.1 ± 1.6	0.68	0.87	0.31
Anxiety	7.7 ± 2.5	7.1 ± 3.2	8.2 ± 2.9	83 ± 2.3	6.8 ± 3.1	8.9 ± 1.7	0.32	0.72	0.22
Depression	8.5 ± 2.3	8.0 ± 2.5	7.8 ± 3.1	8.3 ± 2.2	7.4 ± 2.8	9.0 ± 1.5	0.71	0.30	0.41
Total	129.3 ± 25.2	94.4 ± 30.7	116.3 ± 31.1	133.6 ± 16.5	95.5 ± 27.7	122 ± 17.3	0.38	0.87	0.33

Values are presented as mean ± SD. Abbreviation: Preop; pre-operative, POD1; post-operative 1 day, POD2; post-operative 2 day.
